# Supplementary material for: The interactome of intact mitochondria by cross-linking mass spectrometry provides evidence for coexisting respiratory supercomplexes
Source: Mol Cell Proteomics. 2017 Dec 8;17(2):216–32. doi: 10.1074/mcp.RA117.000470 (PMC5795388; doi:10.1074/mcp.RA117.000470)
Supplement: Supplemental Data [file supp_RA117.000470_133922_0_supp_23978_fzffwf.zip › spectra_annotation/mito_DR_spectra_annotation/43-1-4-1-10-1.pdf]

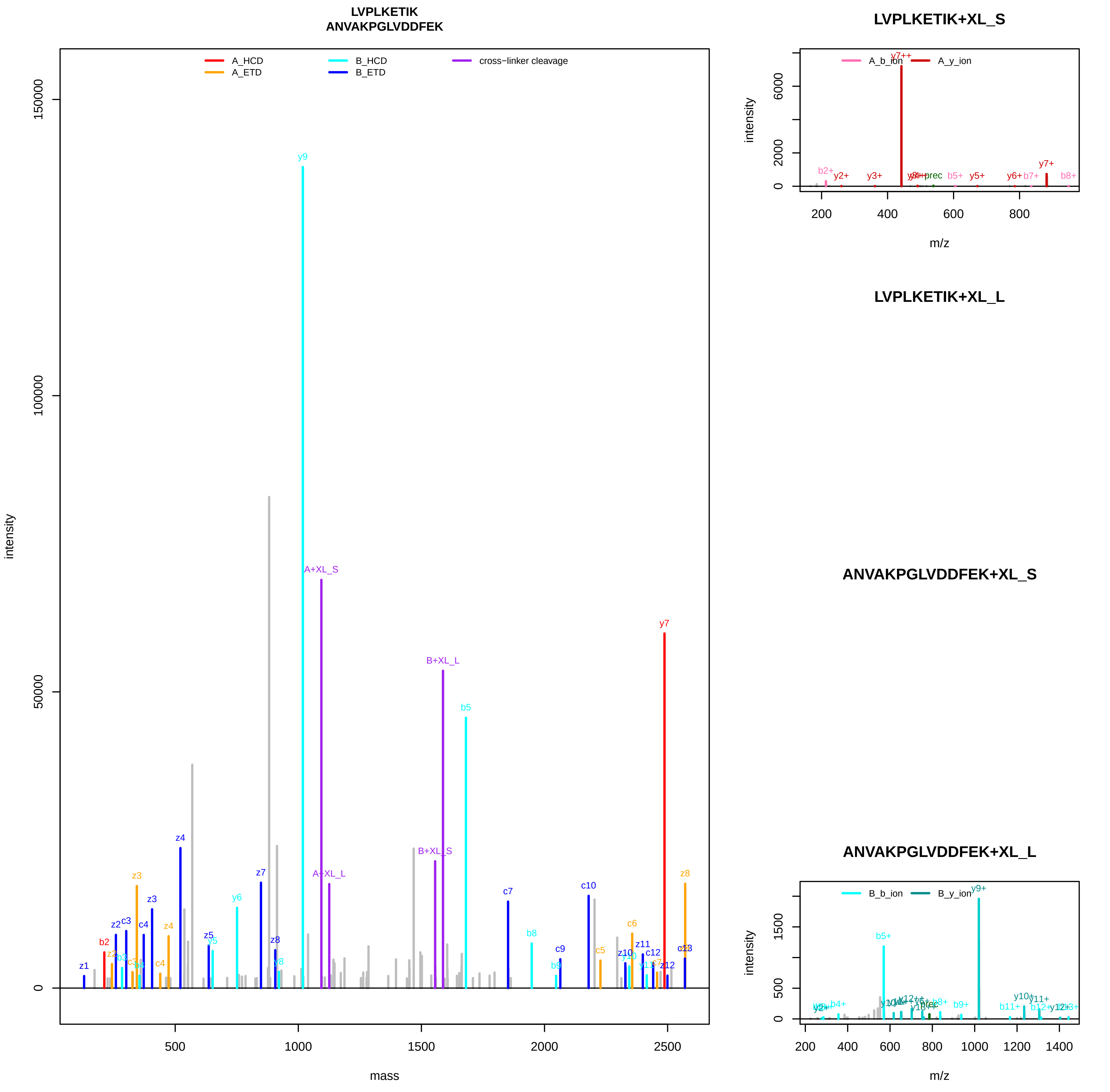

LVPLKETIK
ANVAKPGLVDDFEK
A_HCD
A_ETD
B_HCD
B_ETD
cross−linker cleavage
intensity
mass
0
50000
100000
150000
500
1000
1500
2000
2500
y9
A+XL_S
B+XL_L
B+XL_S
A+XL_L
b5
y7
z8
c13
z12
c12
c7
z11
z10
c6
c5
c10
c9
b9
b8
c7
z7
z8
y8
z5
y5
y6
z4
c4
z3
c3
z2
b2
z1
LVPLKETIK+XL_S
A_b_ion
A_y_ion
y7++
b2+
y2+
y3+
prec
b5+
y5+
y6+
b7+
y7+
b8+
m/z
LVPLKETIK+XL_L
ANVAKPGLVDDFEK+XL_S
ANVAKPGLVDDFEK+XL_L
B_b_ion
B_y_ion
y9+
b5+
b4+
y2+
b8+
b9+
y10+
y11+
b11+
b12+
y12+
